# Plaie iatrogène de l'artère carotide commune secondaire a un cathétérisme veineux jugulaire interne

**DOI:** 10.11604/pamj.2017.26.18.11461

**Published:** 2017-01-16

**Authors:** Cheikh Ahmédou Lame, Birame Loum, Ibrahima Keita, Thierno Boubacar Diallo, Alamasso Sow

**Affiliations:** 1 Service d’ORL et de Chirurgie Cervico-Faciale, Hôpital Principal de Dakar, Sénégal; 2 Service d’Anesthésie-Réanimation, Hôpital Principal de Dakar, Sénégal; 3 Service de Chirurgie Générale, Hôpital Principal de Dakar, Sénégal

**Keywords:** Plaie artère carotide, cathétérisme veineux jugulaire interne, hématome cervical compressif, Carotid artery injury, internal jugular venous catheterization, compressive cervical hematoma

## Abstract

La plaie carotidienne lors du cathétérisme jugulaire veineux est un accident rare mais pouvant être dramatique. Nous rapportons un cas d'hématome cervical compressif avec hémothorax survenu à la suite d'une plaie de l'artère carotide commune au décours d'un cathétérisme veineux jugulaire interne. Le diagnostic et la prise en charge de ce type de complication sont discutés.

## Abstract (en)

Carotid injury occurring during venous jugular catheterization is a rare but potentially dramatic event. We report a case of compressive cervical hematoma associated with haemothorax occurred after common carotid artery injury during internal jugular venous catheterization. The diagnosis and management of this type of complication are discussed.

## Introduction

La plaie de l'artère carotide commune est une complication rare du cathétérisme veineux jugulaire interne. Cette complication peut être gravissime. Nous rapportons un cas d'hématome cervical compressif avec hémothorax survenu au décours d'un cathétérisme veineux jugulaire interne gauche.

## Patient et observation

**Figure 1:**
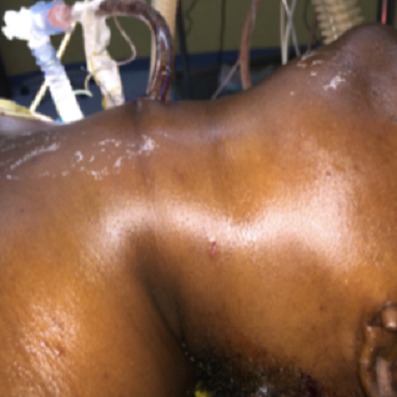
Volumineux hématome cervical ayant imposé une crico-thyrotomie

**Figure 2:**
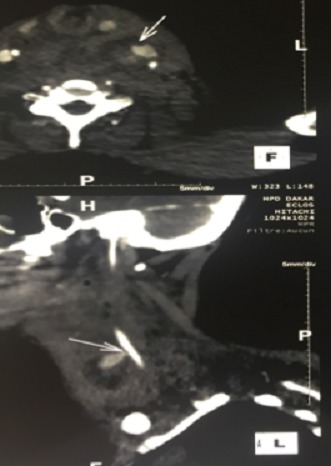
TDM du cou montrant la fuite artérielle carotidienne et le pseudo-anévrysme

**Figure 3:**
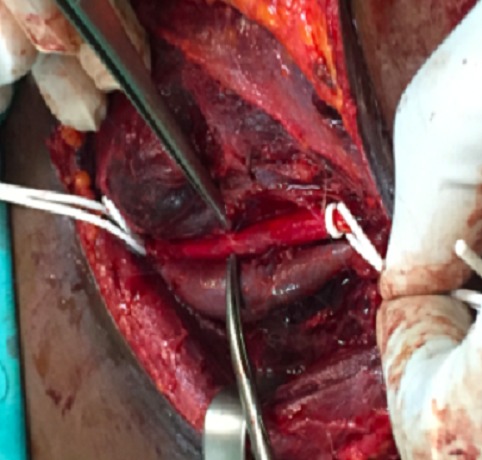
Vue per-opératoire avec la brèche carotidienne commune et l’hématome cervical

Une patiente âgée de 37 ans était admise en réanimation depuis cinq mois pour des brûlures thermiques étendues. Des soins locaux quotidiens étaient réalisés sous anesthésie générale. Une tentative de mise en place d'un cathétérisme veineux jugulaire interne gauche se soldait par un hématome au point de ponction ayant imposé l'ablation du cathéter, un pansement compressif et le changement de site de ponction. Douze heures après ce geste, la patiente présentait une volumineuse tuméfaction cervicale avec détresse respiratoire et signes de choc cardiovasculaire. Une cricothyrotomie était réalisée en urgence (Figure 1) et des mesures de réanimation hémodynamiques mises en place. Le scanner cervico-thoracique, après stabilisation de la patiente, mettait en évidence une brèche de l'artère carotide commune avec un volumineux hématome cervical et un hémothorax important (Figure 2). La patiente était immédiatement admise au bloc opératoire. Une cervicotomie exploratrice retrouvait une plaie carotidienne commune (Figure 3) qui était réparée, suivie de l'évacuation de l'hématome cervical. L´évolution de la patiente était favorable.

## Discussion

Le cathétérisme veineux central est un geste fréquemment réalisé en milieu de réanimation. Il permet de disposer d'un accès veineux de bonne qualité pour le remplissage vasculaire, l'administration intra-veineuse des drogues, l'alimentation parentérale, l'hémodialyse [1–4]. L'incidence de son utilisation en réanimation est estimée à 30% en France [1]. Tandis qu'aux Etats Unis, 15 millions de voies veineuses sont posées chaque année [5, 6]. Cet acte, classique en milieu de réanimation, n'est pas anodin car associé dans 15% de cas à des complications, d´ordre mécanique (5 à 19%), infectieux (5 à 26%), ou thrombotique (2 à 26%) [6–8]. La veine jugulaire interne (VJI) est fréquemment utilisée. La pose de la voie veineuse est souvent faite « à l'aveugle », utilisant des repères ostéo-musculaires de surface [3, 9]. Le rapport intime entre VJI et axe carotidien explique 0,5 à 11,4% des plaies carotidiennes qui surviennent lors du cathétérisme veineux jugulaire interne [1, 2, 6, 8]. Les conséquences de cette plaie sont représentées par le choc hémorragique, le pseudo-anévrysme, la fistule artèrio-veineuse, la dissection et les manifestations compressives notamment respiratoires [2, 3, 9]. Certaines peuvent être dramatiques. Chez notre patiente un hématome immédiat du site de ponction, suivi d'une tuméfaction progressive du cou entraînant une détresse respiratoire et une détérioration hémodynamique ont été les signes révélateurs. L'angio-scanner met en évidence la position intra-arterielle du cathéter lorsque celui ci est laissé en place. Il permet d'objectiver aussi la fuite artérielle le pseudo-anévrysme et/ou l'hématome cervical constitué [2, 8] comme l'illustre la figure 2. Les facteurs favorisant ces complications hémorragiques lors du cathétérisme VJI sont les troubles de l'hémostase, un traitement anticoagulant, la difficulté d'insertion du cathéter (multiples ponctions veineuses et ponction artérielle), un cou court, l'obésité, l'inexpérience de l'opérateur et le contexte d'urgence [2, 4, 6, 10]. Certains auteurs ont montré que l'utilisation du guidage échographique réduit les risques de complications iatrogènes du cathétérisme VJI [1, 3, 5, 6, 9]. Cependant, la disponibilité d'un appareil d'échographie n'est pas toujours effective en milieu de réanimation dans nos structures. Sur le plan thérapeutique, l'ablation du cathéter suivie de la compression manuelle du site de ponction est généralement suffisante chez les patients non anticoagulés [1, 2, 8]. Une surveillance rapprochée est alors nécessaire. Trente pour cent (30%) des patients deviennent symptomatiques après ce geste de retrait/compression (pull and pressure technique). Trente trois pour cent (33%) de ces patients symptomatiques décèdent. Les causes de décès sont la détresse respiratoire et le choc hémorragique [1, 6]. Certains auteurs préconisent l'exploration chirurgicale d'emblée, l'ablation du cathéter avec artériotomie et réparation de l'artère lésée. Cette méthode serait plus sûre et plus efficace dans la gestion des plaies carotidiennes par cathétérisme VJI [2, 3, 6]. Récemment, des techniques de chirurgie endovasculaire avec mise en place de stent ou utilisation de matériel de suture artérielle percutanée ont été décrites [2, 3, 6].

## Conclusion

Le cathétérisme veineux central est un geste usuel en milieu de réanimation. La voie jugulaire interne est fréquemment utilisée. Mais, cette procédure, non anodine, expose dans 15% des cas à des complications immédiates ou différées qui peuvent être graves. Ce cas de plaie de l'artère carotide commune illustre le risque de complication grave du cathétérisme VJI. Une technique rigoureuse de pose et/ou l'utilisation du guidage échographique permettent de minimiser et de prévenir ce risque parfois létal et d'améliorer ainsi la qualité des soins. La prise en charge de cet accident passe par le retrait du cathéter suivi de la compression du point de ponction. L'échec de cette technique conduit à l'abord chirurgical ou à la réparation endovasculaire.
